# Partnering Early to Provide for Infants At Risk of Cerebral Palsy (PĒPI ARC): protocol for a feasibility study of a regional hub for early detection of cerebral palsy in Aotearoa New Zealand

**DOI:** 10.3389/fped.2024.1344579

**Published:** 2024-04-04

**Authors:** Angelica Allermo Fletcher, Gaela Kilgour, Meghan Sandle, Sally Kidd, Alison Sheppard, Stephanie Swallow, Ngaire Susan Stott, Malcolm Battin, Wyllis Korent, Sian A. Williams

**Affiliations:** ^1^ Neonatal Intensive Care Unit, Wellington Regional Hospital, Wellington, New Zealand; ^2^ Queensland Cerebral Palsy Research and Rehabilitation Service, The University of Queensland, Brisbane, QLD, Australia; ^3^ Child Development Service, Wellington Regional Hospital, Wellington, New Zealand; ^4^ Department of Surgery, Faculty of Medical and Health Sciences, University of Auckland, Auckland, New Zealand; ^5^ Newborn Services, Auckland City Hospital, Auckland, New Zealand; ^6^ School of Allied Health, Curtin University, Perth, WA, Australia; ^7^ Liggins Institute, University of Auckland, Auckland, New Zealand

**Keywords:** General Movements Assessment (GMA), Hammersmith Infant Neurological Examination (HINE), early diagnosis (MeSH), health inequity, early development

## Abstract

**Introduction:**

Cerebral palsy (CP) can now be diagnosed in infants with identified CP risk factors as early as three months of age; however, many barriers prevent equitable access to early detection pathways. The “Partnering Early to Provide for Infants At Risk of Cerebral Palsy” feasibility study (PĒPI ARC) seeks to trial a new approach to decrease inequitable health service in Aotearoa New Zealand for high-risk infants and their families. PĒPI ARC incorporates face-to-face clinics, an in-person and virtual Hub, and the use of telehealth to enable flexible access to CP assessments and support for health professionals in early CP detection.

**Methods and analysis:**

A non-randomised feasibility study was conducted from a tertiary Neonatal Intensive Care Unit (NICU) in Wellington and included seven regional referral centres, servicing nearly 30% of the total population in New Zealand (NZ). The families of infants with a high risk of neurodevelopmental impairment and health professionals interacting with the Hub were invited to participate. Mixed methods were used to evaluate the (i) equitable implementation of an early detection pathway, (ii) acceptability, (iii) demand among families and health professionals, (iv) efficacy in relation to reducing the age of receipt of CP diagnosis, and (v) the experiences around communication and information sharing.

**Ethics and dissemination:**

The NZ Health and Disability Ethics Committee approved this study (HDEC: 2022 FULL 13434). The findings will be disseminated in peer-reviewed journals, in conference presentations, and via professional networks.

**Clinical trial registration:**

Australian New Zealand Clinical Trials Registry: ACTRN12623000600640.

## Introduction

In Aotearoa New Zealand (NZ), it is estimated that every 3 days a baby is born who will later receive a diagnosis of Hōkai Nukurangi, the Te Reo Māori term for cerebral palsy (CP) (1). Of these babies, approximately 60% are diagnosed after 12 months of age, with a reported median age of CP diagnosis at 17 months of age in NZ (1). Many families of children with CP living in NZ perceive diagnosis delays, with nearly 20% of them reporting delays of more than 12 months (2).

Historically, CP has been diagnosed using a combination of clinical and neurological signs, which often do not appear until the child is older. In 2017, an international evidenced-based guideline was published for accurate and early CP diagnosis using a combination of different assessment tools (3). Through a combination of brain Magnetic Resonance Imaging (MRI), the Prechtl General Movements Assessment (GMA) (4), and the Hammersmith Infant Neurological Examination (HINE) (5), infants with detectable newborn risk factors for CP can now be diagnosed as early as 3 months of age (corrected for prematurity) with a sensitivity and specificity of 97% and 99%, respectively, in a retrospective case–control study (6). The successful implementation of these recommendations has seen significant reductions in the age of identifying “high risk of CP” [e.g., in an Australian clinic with an average age of 4.4 months (7)] and the average age of CP diagnosis [e.g., in a US clinic from 18 months prior to implementation down to 12 months (8), in a network of US clinics 19.5 months to 9.5 months (9), and in an Australian clinic to 8.5 months (7)].

Based on international recommendations (3, 10), the NZ Cerebral Palsy Clinical Network (CPCN) in 2020 developed the national *Best Practice Recommendations for Early Detection of CP, Intervention, and Monitoring* (CPCN Recommendations) to support the implementation of early clinical CP detection pathways across NZ. The development of the CPCN Recommendations addresses the need to provide clear pathways (11) and guidance for consistent assessment approaches (12), but it does not remove other barriers to implementing early detection in practice. These barriers, as reported by health professionals, include the lack of resources and multidisciplinary teams (12); poor coordination between hospital, community, and regional follow-up; and inequitable access to services (11–13). Families report dissatisfaction and distrust with the diagnostic process, delays in diagnosis, and issues with communication and access to practical and emotional support (2). In response to these concerns, co-design workshops for Māori and non-Māori families were conducted and revealed the need for improved service provision and navigation within a complex health system, with a “one-stop shop” solution proposed as a more equitable and culturally safe system (14). The “Partnering Early to Provide for Infants At Risk of Cerebral Palsy” (PĒPI ARC) Hub seeks to incorporate these learnings within the implementation of the CPCN Recommendations.

### PĒPI ARC

The PĒPI ARC Hub applies a modified multidisciplinary team (MDT) approach to health service delivery and care for infants with detectable risk factors for CP who are admitted to a tertiary neonatal intensive care unit (NICU). The Hub aims to develop a Māori-centered relationship model of care with Whanaungatanga (connectedness) and Whakawhanaungatanga (building relationships) at its core (15), incorporating the key interrelated dimensions of Māori holistic healthcare including wairua (spiritual), whānau (extended family network), hinengaro (the mind, emotion), and tinana (physical). Whānau represents the collective way Māori operate. People do not exist in isolation but include a person's immediate and extended family. PĒPI ARC embraces the view that at the centre of care is the patient (i.e., infant) and their extended family and seeks to (1) build relationships and connections through the journey of infants in NICU, their transition to home, and follow-up support in their community (16) and (2) to coordinate care and minimise the burden for families in attending multiple appointments with different health professionals.

The Hub provides three functions:

1. A face-to-face clinic for infants and their families living in Wellington
2. Support to families and infants who reside in other regions to access GMA and HINE assessments, by working in close partnership with the families, their local doctors, and therapists
3. Partnership with, and support for, local and regional health professionals to use early detection strategies and communication with families

### Study aim, objectives, and hypotheses

This study aims to evaluate the feasibility of equitable implementation of a pathway for the early detection of CP for infants with newborn detectable risks (criteria outlined in Table 1) who are admitted to a tertiary NICU in NZ through a multidisciplinary regional Hub called the PĒPI ARC Hub. Based on the work of Bowen et al. (17), (i) **implementation**, (ii) **acceptability (**18), (iii) **demand,** and (iv) **limited efficacy** of PĒPI ARC will be studied.

**Table 1:** Criteria for infants within the newborn detectable risks, as outlined in the New Zealand CPCN best practice recommendations for early detection of cerebral palsy (CP), intervention, and monitoring (CPCN recommendations).

Newborn detectable risk criteria
1. Any infant born <28 + 0 weeks gestation. OR 2. Any infant born ≥28 + 0 weeks gestation with one or more detectable risk factors for CP: i. Weight < 1,000 g ii. Intrauterine growth restriction Birth weight <3rd percentile on population-based or customised growth charts or at clinician's discretion based on concern about pathological growth restriction. iii. Abnormal findings on neuroimaging associated with CP [e.g., Grade 3 and 4 intraventricular haemorrhage, post-haemorrhagic ventricular dilatation, hydrocephalus, periventricular leukomalacia (PVL), stroke, brain maldevelopment]. iv. Hypoxic ischaemic encephalopathy (HIE)—Grade 2 and 3. v. Neonatal encephalopathy of other aetiology. vi. Neonatal meningitis/ encephalitis—bacterial or viral. vii. Cardiac surgery viii. Clinical or parental concerns or other significant risk factors—clinician discretion.

### Primary objective

1. **Implementation:** to determine to what extent the CPCN Recommendations for early CP diagnosis can be equitably implemented for infants with newborn detectable risks who are admitted to a level 3 NICU (independent of place of residence and ethnicity) and if the fidelity of the CPCN Recommendations can be maintained

### Secondary objectives

2. **Acceptability:** to evaluate the acceptability of a PĒPI ARC Hub model of care by families and health professionals
3. **Demand:** to determine the demand for the PĒPI ARC Hub among both local and regional families and health professionals
4. **Limited efficacy**:
  i. To evaluate if the PĒPI ARC Hub model reduces the age of diagnosis of CP
  ii. To explore the experiences around communication and information sharing between families and health professionals

### Hypotheses

**H1**: Equivalent levels of infants who meet the criteria will undergo assessments with CP-specific diagnostic tools outlined in the CPCN Recommendations, independent of region of residence or ethnicity.

**H2**: Whānau and health professionals will find the PĒPI ARC Hub model of care acceptable (over the acceptability constructs).

**H3**: The demand will be high with infants who meet the criteria choosing to enroll in the PĒPI ARC study over standard care.

**H4**: More than 34% of infants with CP will be diagnosed before 12 months of corrected gestation age (CGA) and >17% before the age of 6 months CGA.

## Methods and analysis

This study protocol was designed according to the Standard Protocol Items: Recommendations for Interventional Trials (SPIRIT) checklist (19), with adaptations based on the recommendations outlined by Thabane and Lancaster (20).

### Study design and setting

The PĒPI ARC study is a prospective, non-randomised, feasibility study that uses a mixed methods approach to evaluate the previously outlined domains of feasibility. The study will run from March 2023 to September 2024, with the primary time point being at the completion of the PĒPI ARC Hub trial period. The study setting will be a regional Hub based in Wellington to allow follow-up of eligible infants who received care at the Wellington NICU. The PĒPI ARC Hub will be delivered using two components: a community-based, multidisciplinary face-to-face diagnostic clinic in Wellington and a virtual Hub to support regional CP-specific neurodevelopmental follow-up, education, and information sharing and foster relationships among the Wellington team, local community, and regional child development services (CDS) and paediatricians.

The Wellington NICU is a tertiary unit that provides perinatal and surgical care for infants from Wellington and seven regional referral centres from half of the North Island and the top of the South Island of NZ. The unit cares for a third of all infants admitted to a tertiary NICU in NZ. It is estimated that between 100 and 120 infants per year will fit the criteria for the CPCN Recommendations pathway, of which approximately 30% live in the Wellington area (21) (i.e., to be invited to attend the in-person clinic), and the remainder will receive follow-up in their local regions (supported by the PĒPI ARC team through telehealth).

For the Wellington NICU catchment area, data from the NZ CP Register (2009–2020) showed that 17% of infants received a diagnosis of CP before 6 months of age and 34% before 12 months (22).

### Patient and public involvement

The development and refinement of the study question, study protocol, Hub design, and resources were guided by three working groups: whānau Hui, the advisory group (provided local and cultural advice), and the steering group. A specific focus was placed on the voices of Māori and Pasifika whānau, who are reported to have higher rates of preterm birth (23) and poorer preterm outcomes (24). In the planning stages of PĒPI ARC, whānau hui (meetings/gatherings with family members of infants who had been admitted to the NICU) involved focused discussions about their Wellington NICU experience, reflections on their journey, use of assessment tools, and possible improvements. Families were informed about the study design in the following areas:

• Early diagnosis conversations and information sharing—consistent, timely, honest, and appropriate
• Service delivery: the experience of early diagnostic assessments in NICU and within the community service
• Hearing the voice of the parent
• Hub design
• Cultural needs and safety

The PĒPI ARC advisory group consisted of families and health professionals including a Māori researcher and advisor to support our commitment to Te Tiriti O Waitangi and equitable health outcomes for Māori and a Pasifika social worker. The PĒPI ARC steering group, formed by experts in early detection of CP from NZ and Australia, guided the planning and provided ongoing oversight of the study.

### Participants and recruitment

Participants for this study will be formed by two groups, namely, the infants and their parent/caregivers, and the health professionals involved/interacting with the PĒPI ARC Hub. There are no limits placed on the sample size, and a formal sample size calculation is not required. Based on the historical figures of the number of infants admitted to the NICU at the Wellington Regional Hospital and infants aged <28 weeks, up to 120 infants are anticipated to meet the study eligibility during the 12-month recruitment period. There will be no restrictions on other care or interventions partaken in by participants.

#### Infants and parents/caregivers

Infants must meet the following eligibility criteria:

i. admitted to the NICU at Wellington Regional Hospital within the study period,
ii. have a discharge address located within the Wellington region or within one of the referral regions of the Wellington NICU, and
iii. and meet the criteria of the CPCN Recommendations for newborn detectable risks for CP (Table 1).

Infants will be excluded if they are born with a life-limiting condition and not expected to survive past the first year of life or if the family is expected to move out of the catchment area before the infant is 3 months CGA.

Infants and their parents/caregivers will be invited to participate in the study by a member of the PĒPI ARC research team (not directly involved in providing healthcare for the infant in the NICU). Recruitment can occur at any point during the NICU admission prior to discharge. Support from social workers and Whānau Care Services is available to assist with recruitment decision-making, and parents are encouraged to discuss and seek support from their extended family. Families will be advised that they can instead choose the usual care option (see below).

#### Health professionals

All health professionals who engage with the PĒPI ARC Hub will be invited to complete a questionnaire about their experience and acceptability of the PĒPI ARC model of care. There are no exclusion criteria. Health professionals will be invited to complete a semi-structured interview about their experiences and acceptability of the PĒPI ARC Hub.

The members of the PĒPI ARC team will complete a team-specific questionnaire focused on the acceptability of PĒPI ARC.

### Intervention—while in the NICU

#### All infants enrolled in PĒPI ARC

All families will receive:

• *Welcome folder* with information about the study and the PĒPI ARC team and documents to help them navigate their NICU journey and transfer to a regional centre or discharge home with community follow-up. They will also be provided with education and instructions on filming and transferring GMA videos to the PĒPI ARC team after discharge.
• *PĒPI passport* aimed to improve health literacy, support a relationship model of care in the NICU, allow families to share neurodevelopmental information about their infant with different healthcare providers after discharge from NICU, and help smooth the transition from the NICU to regional centres.

The passport was developed in partnership with the health professionals and families and includes both English and Te Reo Māori translations. Families can document the developmental strengths and challenges, completed assessments and results, and plan for medical and developmental follow-up of their infants.

#### Infants and families living in the regional referral areas

A PĒPI ARC research nurse will support out-of-region families throughout their NICU journey, upon discharge/transfer, and follow up with the regional services to provide continuity of care as part of the “one-stop shop”. Infants from Wellington will be supported by the discharge facilitator team as part of NICU usual care.

### Intervention—after discharge from the NICU

#### Infants and families living within the Wellington region

##### Face-to-face clinic

A face-to-face MDT clinic appointment (lasting 90 min) will take place in a community setting when the infant is between 12 and 16 weeks CGA. This Hub appointment will replace their usual care appointment with their hospital neonatologist at the same age. The MDT will consist of a neonatologist or developmental paediatrician, a GMA and HINE-trained therapist, a social worker, and a clinic nurse and may also include assessments by a speech and language therapist (SLT)/dietician if clinically indicated.

The infant will have a growth, development, medical, and social work assessment, and will be evaluated using the Feeding Matters Infant and Child Feeding Questionnaire (ICFQ) (25). Any previously collected neuroimaging and GMA will be reviewed, and a GMA and HINE (recording scores and number of asymmetries) will be performed (Table 2). The family will also have time with the social worker in private. Assessment findings, resources, and support will be shared with the family as part of the appointment (as outlined in Table 3) and with the local healthcare providers of the infant via an electronic assessment report. Both the family and health provider will also receive a detailed clinic letter within 2 weeks.

**Table 2:** PĒPI ARC clinic assessment schedule (12–14 weeks CGA).

Assessment	Schedule	Trigger for second clinic visit/referrals
Neuroimaging head ultrasound/MRI	Review of prior neuroimaging and need for further imaging assessed.	Referral for MRI (if not already obtained) if: • Abnormal/absent GMA and/or • HINE score < 57, and assessed at high-risk of CP
GMA	• General Movements Assessment of presence or absence of normal fidgety movements. • Review of video captured by parents/caregivers prior to clinic, or video taken at clinic visit^a^	Repeated 2 weeks later if abnormal/absent
HINE	• 3 months Hammersmith Infant Neurological Examination	Recommended to repeat at 6 months CGA if GMA and HINE assessments are incongruent.
ICFQ (25)	• Completed by the family before or at the clinic appointment	Referral to speech and language therapist (SLT) and/or dietician will be made (if not already involved) if two or more questions indicate red flags for feeding difficulties (25)

MRI, Magnetic Resonance Imaging; GMA, General Movements Assessment; HINE, Hammersmith Infant Neurological Examination; CP, cerebral palsy; CGA, corrected gestational age; ICFQ, The Feeding Matters Infant and Child Feeding Questionnaire.

^a^ Scored by the GMA team or at least two senior assessors (inter-rater reliability of the assessors will be established beyond 80% prior to PĒPI ARC clinic appointments beginning). If consensus on scoring is not reached, the GMA will be reviewed independently by a third assessor, including referral to expert assessors if needed until consensus is reached.

**Table 3:** Process plan for communicating outcomes to families/health professionals.

If an infant is assessed to be at low risk of CP: • Verbal and written feedback will be provided to the family and local team. • The infant will be discharged from the PĒPI ARC clinic to usual care.
If the assessments are inconclusive: • Explanation of inconclusive findings will be provided verbally and documented in clinic letters provided to the family and neonatologist/therapist. • The infant will be seen in the PĒPI ARC clinic for further assessment in 2–4 weeks for a repeat clinical examination.
If an infant is diagnosed with CP or high-risk of CP: • The family will be offered a follow-up appointment with the PĒPI ARC team including the social worker within 1–2 weeks’ time (face to face or telehealth based on their preference) to answer any questions and receive more information. All ongoing care will be with their local neonatologist/paediatrician and therapist. • Screening for comorbidities will occur, and genetic testing will be offered. Support and follow-up referral will be made. • Information about the diagnosis of high-risk of CP or CP, the referrals made, and advice about ongoing follow-up and monitoring will be provided to the family of the infant, primary neonatologist/paediatrician, therapist, and GP.

CP, cerebral palsy; GP, general practitioner.

##### Telehealth

Local families unable to attend the clinic in person will be offered their MDT assessment using telehealth with assistance from the therapist or clinic nurse. The HINE assessment will be arranged to be completed by the PĒPI ARC therapist either at the time of the telehealth appointment or at another time convenient for the family. They can also contact the Hub/PĒPI ARC team via email and phone at any time within the study time to request support or answer questions.

#### Infants and families living outside of the Wellington region

##### Virtual hub

The virtual PĒPI ARC Hub will offer flexible support for families, paediatricians, and therapists to aid the timely completion of early detection assessments (Table 2). The Hub will be able to assist with the interpretation of specific assessments, triangulation of the results, CP diagnosis, and communicating the assessment outcomes to the families. If an infant receives a high risk of CP/CP diagnosis, additional information relating to early intervention, monitoring of comorbidities and complications of CP, parental support, and parent information about CP will be offered.

### Usual care for infants at high risk of neurodevelopmental impairment admitted to the Wellington NICU

#### All high-risk infants

• MRI is routinely done only for infants with hypoxic ischaemic encephalopathy (HIE) Grades 2 and 3.
• Routinely referred to the neonatal therapist of the unit and on an as-needed basis to other allied health.
• Whilst staff try to implement the CPCN Recommendations, early GMA assessments are not always completed due to medical status and availability of the infant.

#### Infants from the Wellington region

• Followed by the discharge facilitator team throughout their NICU admission and for a period after discharge. This team provides emotional and practical support, prepares families for discharge, organises family meetings, ensures follow-up appointments are made, and assists families once home with feeding support including nasogastric tube feeding and assessment of infants between clinic visits.
• Referred to a visting neonatal therapist on discharge for neurodevelopmental surveillance, who may assist with GMA and HINE. The onset of this service and the length of follow-up time may vary.
• Followed-up by their hospital neonatologist, variable number of appointments, and length of follow-up.
• Follow-up with SLT, dietician, and other health professionals only if referred prior to discharge.
• No social work follow-up.
• No MDT clinics.

#### Infants from outside the Wellington region

• There is no service similar to the discharge facilitator team for infants whose families reside outside of Wellington during their NICU stay.
• Referred for paediatric and CDS for follow-up and neurodevelopmental surveillance on discharge from NICU. Variation between regions for the onset of services, length of follow-up time, and number of appointments and assessments (26).
• Three regional centres have clinicians that trained in GMAs, and a further three regional centres send GMA videos to the Wellington GMA team for scoring.
• All regions have therapists trained in HINE. The timing of HINE assessments varies.
• An electronic report with individual GMA scores and an impression of outcome based on the trajectory at 3–4 months CGA is forwarded by the Wellington GMA team to a local therapist following the evaluation of scores.
• No MDT clinics.
• It is important to acknowledge that during the study timeline, changes in “usual care” may occur.

### Outcome measures and data collection

Feasibility will be assessed by using the outcomes, measures, and data capture summarised in Table 4, from March 2023 to September 2024. Data will be directly entered into REDCap (hosted by the Wellington Hospital and MRINZ infrastructures), which will only be accessed by the members of the research team involved in the data entry and analysis. Due to the scope and minimal risks associated with the study, there is no formal data monitoring committee. Interim data monitoring will be conducted at a 6-month review of survey feedback, and auditing of trial conduct will be completed by the primary investigator and study and clinical coordinators on a fortnightly basis. Quantitative data collection tools, surveys for families, health professionals and the PĒPI ARC MDT team, participant information, consent form, and data management plan are available on Open Science Framework (OSF, DOI: https://doi.org/10.1101/2023.10.31.23297869).

**Table 4:** Summary of outcomes, data capture, and measures/approaches for PĒPI ARC.

Objective	Data capture	Measures/approaches
Implementation		
1. To determine to what extent the early CP detection assessments can be implemented equitably to infants with newborn detectable risks admitted to a level 3 NICU	• Demographic information for infants eligible for the CPCN Recommendations pathway (NICU discharge data) • PĒPI ARC clinic notes and medical records	The proportion of infants completing the neuroimaging, GMA, and HINE (as per CPCN Recommendations), with outcomes grouped and compared by: • Region of residence • Ethnicity (Māori, Pasifika, non- Māori/Pasifika)
2. To evaluate if the fidelity of the CPCN recommendations can be maintained, including diagnostic assessments completed, communication with families, and related referrals	• Infant medical records/PĒPI ARC clinic notes • Records for assessments • Referral data • Questionnaire—health professionals • Semi-structured interviews with health professionals and families	The total number and proportion of diagnostic tests completed/not completed (i.e., not grouped by region or ethnicity). Of participants with a high risk of CP/CP detected: • The number of participants whose parents were informed of the diagnosis, and the age and acceptability of communication • The timing of therapist review post high-risk CP diagnosis • The timing of referral for surveillance of comorbidities and medical complications as indicated • The number of families receiving information about practical and emotional support
Acceptability		
3. To evaluate the acceptability of the PĒPI ARC Hub model of care by families and health professionals	• Questionnaires—families, health professionals, and the PĒPI ARC team. • Semi-structured interviews with families and health professionals	Data will be interpreted according to those families and health professionals involved in the Hub who were from Wellington compared with other regional centres
Demand		
4. To determine the demand for the PĒPI ARC Hub among families and health professionals	• PĒPI ARC clinic notes/statistics • Infant medical records (virtual Hub) • Communications with the PĒPI ARC team: logged as part of an audit trail. The full audit trail will be reviewed at the end of the study in combination with questionnaire data	Using the number of infants and families approached to take part in PĒPI ARC: • The proportion of families who consent to the study • The proportion of infants who attend the clinic • The proportion of infants who complete regional follow-up appointments with paediatricians/therapists • The proportion of families that chose to use telehealth • The number and profiles of health professionals accessing the Hub for support • The type of support requested by families and health professionals
Limited efficacy		
5. To evaluate if the PĒPI ARC Hub model reduces the age of CP diagnosis	• PĒPI ARC clinic notes • Infant medical records	• The average age when a preliminary high-risk CP or CP diagnosis was made and age (/rate) of CP confirmation
6. To explore the experiences around communication and information sharing between families and health professionals	• Semi-structured interviews with families and health professionals	• Qualitative analysis of participant experience

NICU, neonatal intensive care unit; CP, cerebral palsy; GMA, General Movements Assessment; HINE, Hammersmith Infant Neurological Examination.

#### Clinic/medical data

Quantitative data will be collected from medical records and PĒPI ARC clinic notes and through communication with local and regional health professionals. Data will include demographic and ethnicity information, indication for entry into the CPCN Recommendations pathway, and completion of assessments including timing and results, diagnostic outcomes, and referrals for monitoring and interventions.

Descriptive statistics will be used to report participant characteristics, recruitment rates, attendance rate in clinics, completion of diagnostic tools, referral for early intervention, and surveillance (Objectives 1, 2, 4, and 5, Table 4). Analysis of Likert scale data from family, health professional, and PĒPI ARC team questionnaires will also be analysed using descriptive statistics (Objective 3, Table 4). If data are determined to be normally distributed, then continuous data results will be reported using means and standard deviations, or if data are not normally distributed, then median and interquartile ranges will be reported (lower and upper quartiles). Pearson's chi-squared or Fisher's exact test will be used to compare the extent of implementation of the best practice guidelines between regions and ethnicities. A *p* value of <0.05 will be considered statistically significant.

#### Audit/records of communications (to health professionals and families)

Communication between the PĒPI ARC Hub, families, and health professionals will be captured via notifications to the PĒPI ARC email (Objective 4, Table 4). These will be filed in the inbox according to themes throughout the duration of the study and then reviewed and analysed after study completion.

#### Questionnaires

Questionnaires will provide quantitative and qualitative information. Questions were developed based on the theoretical framework of acceptability to evaluate the following constructs of acceptability: affective attitude, burden, ethicality, intervention coherence, opportunity costs, perceived effectiveness, and self-efficacy (18).

Families will be sent a link of the anonymous questionnaire within 2 weeks of attending the PĒPI ARC clinic or at 5–6 months CGA if their infant is followed up out in their local region. Display logic is embedded within the questionnaire to adjust the line of questions relating to in-person vs. virtual Hub attendance. All families will be asked to reflect on their experiences of communication, resources, and support (Objective 3, Table 4).

Health professionals attending a PĒPI ARC clinic and who are not members of the regular PĒPI ARC team will be sent a survey within 2 weeks of the clinic appointment. Other health professionals not part of the PĒPI ARC team who have engaged with the Hub (through email audit trail review and being identified as being a primary person involved in the care of an infant) will be sent a survey invitation between 6 and 12 months of PĒPI ARC operating (Objective 3, Table 4).

The members of the PĒPI ARC team will complete a specific team questionnaire focused on the acceptability of PĒPI ARC 3 months after clinic commencement. The data from the team questionnaire will be analysed in real time (i.e., within 4 weeks of capture) to allow for any changes to the study conduct if indicated. The questionnaire will be repeated at the study endpoint to reassess for potential changes for service improvements (Objective 3, Table 4).

Full analysis of all questionnaire data will occur at the end of the study period.

#### Semi-structured interviews

Semi-structured interviews will seek to capture the acceptability of PĒPI ARC (18) in addition to the experiences around communication of the diagnostic outcome, information sharing, and resources and emotional supports provided (Objective 6, Table 4). Questions will target the experiences of implementing the CPCN Recommendations within local and regional settings for health professionals and support provided by the PĒPI ARC team. A qualitative descriptive approach will be used to understand the experiences of whānau and health professionals in the unique PĒPI ARC Hub context of healthcare delivery. All semi-structured interviews will be offered to take place in a mode of the participant's preference (face to face at the site of the clinic Hub or via videoconference). All interviews will be led by a researcher who is experienced in qualitative interviewing and who has not been in direct contact with the participant as part of the PĒPI ARC Hub and will be audio recorded for transcription.

Initially, all families who have not opted out of being interviewed will be sent an email invitation once their infant is over the age of 6 months CGA. A staged review of recruitment of interviewees will occur after 6 months to ensure that a cross section of infants has been captured, i.e., regional and local, ethnic diversity especially representation of Māori and Pasifika, term and preterm infants, and with/without likely diagnosis of CP. If indicated, purposive sampling will then be used to target underrepresented participant groups.

Health professionals who have had interactions (i.e., received PĒPI ARC clinic letters and GMA reports, email enquiries, and education sessions) with PĒPI ARC over the first 12 months, but are not part of the PĒPI ARC team, will be invited to take part in an interview via email. They will be identified from attendance at in-person clinics or Zoom encounters, audit trail of emails, and PĒPI ARC education/training sessions.

Interviews will be conducted until a saturation of experiences has been reached (i.e., no new experiences are introduced within the interviews) or a wide range of participants and experiences have been collected to ensure “information power” (27). The diversity of participants will be reviewed monthly thereafter, and targeted recruitment may be used. Qualitative thematic analysis will occur separately for family and health professional data but will occur concurrently to help determine any crossover of themes following the methods outlined by Braun and Clarke (28). The themes will be interpreted and discussed and help inform the quantitative data.

## Ethics and dissemination

This study was approved by the NZ Health and Disability Ethics Committee (HDEC: 2022 FULL 13434) and is registered on the Australian NZ Clinical Trials Registry (ACTRN12623000600640). Any modifications to the study protocol will be submitted as an amendment to the ethics committee, and an update will be provided to the ACTRN registration. Informed written consent will be obtained from participants, and data will be de-identified for reporting. Health professionals completing anonymous questionnaires will indicate their consent by questionnaire completion, and those completing interviews will provide written consent. The results of the study will be published and disseminated [co-authored by investigators meeting the authorship criteria of the International Committee of Medical Journal Editors (ICMJE)] in conference abstracts and presentations, peer-reviewed articles in scientific journals, the PĒPI ARC community, organisation newsletters, and media releases. A de-identified dataset may be made available upon reasonable request but may be restricted by ethical limitations.

## Discussion

This paper details the protocol for a feasibility study to ascertain if a multidisciplinary regional Hub, namely, PĒPI ARC, can support the equitable implementation of the NZ CPCN *Best Practice Recommendations for Early Detection of CP, Intervention, and Monitoring* for infants with newborn detectable risks. Feasibility will be assessed for acceptability and demand among families and health professionals and for efficacy in relation to reducing the age of CP diagnosis and improving experiences around communication and information sharing between families and health professionals.

The study is limited to only infants with newborn detectable risk factors for CP who are admitted to the Wellington NICU and who either live in Wellington or in one of its referral regions. As there is no control group, our “usual care” infants with the same risk factors who decline to participate will not be followed and therefore cannot be compared to the PĒPI ARC participants. Economic analysis of the effectiveness of PĒPI ARC for families and the health system will not be conducted.

The study has many strengths. One is the delivery of the Hub using two parts: an MDT face-to-face clinic and a regional virtual approach provides an opportunity to decrease inequitable access to care for local and regional infants and families. Some regional centres may not have the experienced staff to deliver the CPCN Recommendations, and the Hub will support and educate clinicians to ensure regional centres can, over time, deliver the recommended assessments and follow-up. The inclusion of qualitative feedback from both families and health professionals will provide depth of context to our findings.

## Strengths and limitations of the study

• The New Zealand (NZ) best practice recommendations for early detection of cerebral palsy (CP) are based on international guidelines and have been peer reviewed for the Aotearoa NZ context.
• Local and regional health professionals have collaborated to inform the PĒPI ARC protocol with the aim to improve access to early CP assessments and early detection rates of CP.
• Reduction in health inequities for Māori and Pasifika have been targeted through informed partnerships.
• Resource development and planning of PĒPI ARC Hub has been co-designed with families and recognises the ecological context of Aotearoa NZ.
• A limitation is that only high-risk infants with “newborn detectable risks” will be included in the study.

## Author's note

The recruitment for the PĒPI ARC Hub opened to eligible participants in March 2023, with the first participant seen in the face-to-face clinic in June 2023. Participants will continue to be recruited until the end of February 2024 with the trial end date planned for September 2024. Follow-up data will continue to be collected until all enrolled infants have reached 12 months CGA.
